# Pneumonia

**Published:** 1907-05

**Authors:** 


### PNEUMONIA.

W. Parker Worster asserts that it is most notable that pneumonia yields with promptitude to treatment intelligently applied at an early stage of the attack.

Even if called late, the physician can readily save the patient by proper management. His best efforts, however, will be futile unless he recognizes the fact that it is not a lung disease, but an infection with its chief manifestation in the lung dependent upon a toxemia. His article especially concerns the disease in children, and he states that nature has provided its own simple and effective remedy for pneumonia—water. The toxic agent circulating in the blood in pneumonia produces a toxemia, this toxemia spends its full force upon the nerve centers, and the object of treatment is to bridge over the danger, arising from the failure of the vital functions, by enhancing the patient's vital powers until the life period of the diplococcus is terminated, which is about seven days. The cold bath comes to the rescue, stimulating the nerve centers, which govern the functions upon whose capacity the patient depends to withstand the toxic agents circulating in the blood. The mere lowering of temperature is a secondary consideration. In the early stages of the disease he gives the full bath, beginning when the temperature reaches 102.5 with a full bath, at 95° every four hours, reducing the temperature of each bath two degrees until it reaches 80°, but not going below 80° with due regard to the effect produced. With the bathing there should be plenty of good hard thermic friction, which is the sine qua non in all cold baths. If dyspnea, somnolence, stupor or delirium should occur, the child is held in water at 100°, deep enough to cover its hips, for five minutes; after which several basins of water at 70° are poured from high up (if preferred, the operator standing upon a chair) above the shoulders. This can be repeated every two hours if necessary, but it is seldom required, for the effect is magical. The patient is aroused to consciousness, inspiration is deepened and the nervous system is refreshed as by no other remedial agent. This procedure should be used when any head symptoms make their appearance.—Medical Record, February 15th, 1907.

